# Syphilis and human immunodeficiency virus infections among pregnant women attending antenatal care clinic of Gondar family guidance association, Northwest Ethiopia: implication for prevention of mother to child transmission

**DOI:** 10.1186/s12978-019-0691-z

**Published:** 2019-03-04

**Authors:** Belete Biadgo, Ahmed Hassen, Mekuriaw Getaneh, Habtie Tesfa, Kefyalew N. Jaleta, Tegegne Eshetu, Dessie Kasew, Mulugeta Melku

**Affiliations:** School of Biomedical and Laboratory Sciences, College of Medicine and Health Sciences, University of Gondar, P.O Box 196, Gondar, Ethiopia

**Keywords:** Human immunodeficiency virus, Pregnant women, Seroprevalence, Syphilis

## Abstract

**Background:**

Sexually transmitted infections constitute a major public health problem worldwide. Syphilis and HIV infections cause various adverse pregnancy outcomes. Therefore, the aim of this study was to determine the seroprevalence of HIV and syphilis infections among pregnant women at Gondar Family Guidance Association clinic, northwest Ethiopia.

**Methods:**

A retrospective study was conducted using sociodemographic and laboratory data obtained from registration books of Gondar Family Guidance Association clinic from January 2011 to April 2015. A binary logistic regression model was fit to identify factors associated with HIV and syphilis infections. Odds ratios with 95% confidence intervals were calculated to determine the strength of association between factors associated with HIV and syphilis infections. A *p*-value ≤0.05 was considered statistically significant.

**Results:**

A total of 3504 pregnant women were included in the study from January 2011 to April 2015. The seroprevalence of HIV, and syphilis were 145 (4.1%) and 66(1.9%), respectively. Twenty-three (0.66%) women were co-infected. Age group 20–29 years (AOR: 3.86; 95% CI: 1.36–10.89), age group ≥30 years (AOR: 6.08; 95% CI: 2.04–18.14) compared to age < 20 year, and HIV-infection (AOR: 14.6; 95% CI: 8.49–25.18) were significantly associated with syphilis infection. There was a decline in trend seroprevalence of HIV from 5.2% in 2011 to 2.1% in 2015; and decline in syphilis seroprevalence from 2.6% in 2011 to 1.6% in 2015 but not statistically significant.

**Conclusion:**

The data showed that syphilis and HIV infections are still critical public health concerns among pregnant women. Screening of all pregnant women for these infections is valuable. Further community-based studies to identify risk factors are necessary.

## Plain English Summary

Sexually transmitted infections are a common public health problem in Ethiopia. The existence of Human Immunodeficiency Virus (HIV) and syphilis infections during pregnancy poses major health risks to the fetus and the mother. We collected socio-demographic characteristics, laboratory test results data retrospectively from January 2011 to April 2015 and the data were analyzed using SPSS version-20 software. The prevalence of HIV and syphilis infections was determined among pregnant women attending antenatal clinic from January, 2011 to April, 2015.

Three thousands five hundred four (3504) pregnant women were enrolled in this study. The prevalence of HIV was 145(4.1%), and syphilis was 66 (1.9%) of pregnant women. Twenty three (0.66%) women were HIV-syphilis co-infected. Age group 20–29, ≥ 30 years and HIV infection were significantly associated with syphilis infection. The prevalence of HIV declined from 5.2% in 2011 to 2.1% in 2015 and decline in syphilis prevalence from 2.6% in 2011 to 1.6% in 2015.

Syphilis and HIV infections are still a problem among pregnant women in Ethiopia. Screening of all pregnant women during Antenatal Care (ANC) follow up is valuable. Implementing focused and targeted sexual transmitted diseases (STDs) prevention strategies is advisable to decrease the magnitude of HIV and syphilis in the community.

## Background

Sexually transmitted infections (STIs) are amongst the world’s most widespread diseases particularly in low income countries [1]. Syphilis is a systemic disease caused by *Treponema pallidum.* The infection can be classified as congenital or acquired, transmitted through unsafe sexual intercourse or blood transfusion, and it is a cause of adverse pregnancy outcomes, such as fetal loss, and premature delivery [2]. In 2012, it affected nearly one million pregnant women; 350,000 had adverse pregnancy outcome worldwide. Moreover, 16% of women with syphilis had no access to antenatal care (ANC), which accounted for 22.1% of adverse pregnancy outcomes [3]. However, since the beginning of the global initiative for eradication of congenital syphilis in 2007, there was 38% decline in the number of syphilis infections and congenital syphilis [3]. Human Immunodeficiency Virus (HIV) and syphilis are both STIs that remain the major public health problems. The two diseases share some similar risk factors and mode of transmissions such as unprotected sex, blood transfusion and sharing sharp materials [4].

Studies have shown that syphilis infection is a cause of genital ulcers, creating a site for HIV entry and shading due to disruption of the natural mucosal and epithelial barriers integrity. Moreover, by activating immune cells and raising viral load, syphilis could facilitate HIV transmission [5, 6].

In Africa, the overall syphilis prevalence in pregnant women ranges from 4 to 15%; and untreated early syphilis will result in a stillbirth in 25% of pregnancies and death in 14% of newborns [7]. A study across 43 sub-Saharan African countries have shown that adverse outcomes such as stillbirth, neonatal death, low birth weight, and congenital syphilis occur in an estimated 206,000 pregnancies each year [8]. Studies in Gondar, Ethiopia, reported that syphilis and syphilis-HIV co-infections were ranging from 1 to 11.2% [9, 10], and 0.5 to 2.2% [9–11], respectively.

Pregnant women infected with HIV have higher risk of adverse pregnancy outcomes [12]. An estimated 24% of postpartum deaths are due to HIV-infections [13]. Globally, about 240,000 children were newly infected with HIV in 2013, where 90% of infection occurred in sub-Sahara Africa and a large proportion of new infections were the result of mother-to-child transmission (MTCT) [14].

The Ethiopian government has started the prevention of mother to child transmission (PMTCT) acceleration plan in 2012, followed by the national strategy for elimination of MTCT in 2013. Despite these efforts, a significant loss of women and children at every step of the PMTCT cascade is observed [15]. A study revealed that, detection and treatment of infected women with gravidity prior to the 3rd trimester of pregnancy enables prevention of adverse pregnancy outcomes [16]. Voluntary HIV counseling and testing in the context of ANC serves as an entry point for targeted PMTCT.

A few previous studies in Ethiopia have shown the prevalence of HIV (10.33, 11.2%) [10, 11] and syphilis (2.9, 3.7%) [10, 11] but there is no overall national comprehensive report on the epidemiology of syphilis and HIV, local studies concerning these infections are low in number. Screening pregnant women for sexually transmitted diseases (STDs) could provide important clues to control these diseases in the population. Routine screening for STDs is the most cost effective method to prevent MTCT of HIV and syphilis. Lack of awareness about the impact of HIV and syphilis and the extent of the problem are some of the barriers to prevent transmission.

### Objective of the study

#### General objective

- To assess the seroprevalence of syphilis and HIV infections among pregnant women at Gondar Family Guidance Association ANC clinic, northwest Ethiopia.

#### Specific objectives

- To determine the seroprevalence of syphilis infections among pregnant women
- To determine the seroprevalence of HIV infections among pregnant women
- To determine the trend in seroprevalence of syphilis and HIV infections among pregnant women over time.

## Methods and materials

### Study area, design period and population

Gondar Family Guidance Association Clinic is one of the Family Guidance Associations of Ethiopia, a service delivery point in Amhara regional state, established for family planning services and to test innovative approaches to meet the growing demand for modern family planning and other sexual and reproductive health services targeting under-served and marginalized segments of the population. A retrospective study was conducted on 3504 pregnant women who attended ANC follow-up at Gondar Family Guidance Association clinic, northwest Ethiopia from January 2011–April 2015.

#### Inclusion and exclusion criteria

All pregnant women were eligible for the study. Women who had full documentation in the registration book were included, whereas women who had incomplete data like age, laboratory test results, and duplicate records (if one pregnant woman came twice or more at different time) were excluded from the study.

### Laboratory diagnosis and quality control

Antenatal care service provides routine laboratory diagnostic tests for all pregnant women. Five milliliters of venous blood samples were collected by sterile vein puncture procedure three drops of whole blood were used for ABO and Rh blood grouping and then the blood was centrifuged for 5 min at 300 revolution per minute and the serum sample was used for HIV and syphilis screening. Serology tests such as HIV screening, syphilis testing and ABO blood group testing are important tests that are being done during the first ANC visit. HIV testing was done according to the national algorithm recommended by the Federal Ministry of Health of Ethiopia. Rapid HIV tests: HIV (1 + 2) rapid test strip (KHB Shanghai Kehua Bio-engineering Co, LTD, Shanghai, China) as the screening test; and Stat-Pak (Chembio Diagnostic Systems, Inc., New York, NY, USA) as a confirmatory test for positive samples; and Uni-Gold™ (Trinity Biotech Plc, Bray, Ireland) as a tie-breaker test were run. These HIV testing methods were immuno-chromatographic assays. All samples with non-reactive results to KHB were considered negative. Syphilis seroreactivity was tested using the rapid plasma reagin (RPR) test (Human GmbH-Wiesbaden, Germany). All RPR positive sera/plasma were further confirmed by *Treponema pallidum* hemagglutination (TPHA) test (Guangzhou Wondfo Biotech Co, Ltd., Guangzhou, People’s Republic of China). Those pregnant women who tested positive for both RPR and TPHA were diagnosed to have syphilis infection. ABO and Rh blood groups were determined using commercially prepared monoclonal antisera (anti-A, anti-B and Anti-D) antibodies. Laboratory testing was carried out according to the manufacturers’ instructions, and all tests were run using quality controls according to standard operating procedures.

### Data collection and analysis

Data on socio-demographic variables and laboratory test results were collected from pregnant women’s registration books. Data were then cross-checked for completeness, and entered into SPSS version 20software for analysis. Descriptive statistics were performed, and the results were presented in tables. Both bivariate and multivariable binary logistic regression models were fit to identify factors associated with HIV and syphilis infections. Variables with P- value < 0.2 in the bivariate analysis were considered for multivariate analysis to control the possible effect of confounding. Both crude odds ratio (COR) and adjusted odds ratio (AOR) with the corresponding 95% confidence interval (95%CI) were used to determine the strength of the association. A *P*-value *≤*0.05 in the multivariable binary logistic regression analysis was considered to be statistically significant.

## Result

### Socio-demographic characteristics, blood group, syphilis and HIV test status of the study participants

A total of 3504 pregnant women were enrolled from January 2011 to April 2015. About 737 (21%) and 2145 (61.2%) of the study participants were in the age group of < 20 and 20–29 years, respectively. The median (interquartile range) age of the study participants was 25 years (interquartile range: 21–28 years). Almost all, 3359 (95.9%) blood group of the study participants were Rhesus (Rh) positive. According to the year of diagnosis, about 871(24.9%) in 2011, 811 (23.1%) in 2012, 760(21.7%) in 2013, 635(18.1%) in 2014 and 427(12.2%) in 2015 were enrolled (Table 1).

**Table 1:** Socio-demographic characteristics, blood group, and Syphilis and HIV test status of the study participants at the Ethiopian Family Guidance Association Clinic, Gondar, Northwest Ethiopia, from January 2011 to April 2015, (*N* = 3504)

Variables	Frequency (*N* = 3504)	Percentage (%)
Age group in year		
< 20	737	21.0
20–29	2145	61.2
30–39	580	16.6
≥ 40	42	1.2
Syphilis status		
Negative	3438	98.1
Positive	66	1.9
HIV Sero-status		
Negative	3359	95.9
Positive	145	4.1
HIV-Syphilis co-infection(*n* = 145)		
Yes	23	15.9
No	122	84.1
ABO blood group		
A	984	28.1
B	831	23.7
AB	228	6.5
O	1461	41.7
Rh blood group		
Rh positive	3237	92.4
Rh negative	267	7.6

### The seroprevalence and associated factors of HIV infection

The overall seroprevalence of HIV was found to be 145(4.1%) (95% CI: 3.6–4.9). The risk for HIV infection was higher among women aged 20–29 years followed by 30–39 (Table 2).

**Table 2:** Associated factors for HIV sero-positivity among pregnant women attending at the Ethiopian Family Guidance Association Clinic, Gondar, Northwest Ethiopia, from January, 2011 to April 2015, (N = 3504)

Variables	HIV Sero-positive		COR (95%CI)
	Positive	Negative	
Age group in year			
< 20	30	707	0.55 (0.16–1.89)
20–29	84	2061	0.53 (0.16–1.75)
30–39	28	552	0.66 (0.19–2.27)
≥ 40	3	39	1.00
ABO blood group			
A	41	943	0.948 (0.467–1.922)
B	34	797	0.930 (0.452–1.912)
O	60	1401	0.934 (0.471–1.851)
AB	10	218	1.00
Rhesus blood group			
Rh Negative	9	258	1.00
Rh Positive	136	3101	1.257 (0.633–2.498)

*CI* Confidence Interval, *COR* Crude Odds Ratio, *HIV* Human Immunodeficiency Virus

### The seroprevalence and associated factors of syphilis infection

The overall seroprevalence of syphilis was 66(1.9%) (95% CI: 1.4–2.3). The risk for syphilis infection was higher and positively associated among women aged 20–29 years (AOR: 3.86; 95% CI: 1.36–10.89), women aged ≥30 years (AOR: 6.08; 95% CI: 2.04–18.14) and women infected with HIV (AOR: 14.6; 95% CI: 8.49–25.18). Syphilis-HIV co-infections were present in 23 (0.66%) women (Table 3).

**Table 3:** Associated factors for Syphilis sero-positivity among pregnant women attending at the Ethiopian Family Guidance Association Clinic, Gondar, Northwest Ethiopia, from January, 2011 to April, 2015 (*N* = 3504)

Variables	Syphilis Sero-status		COR(95%CI)	AOR(95%CI)	*P* –Value
	Positive	Negative			
Age group in year					
< 20	4	733	1.00	1.00	
20–29	42	2103	3.66 (1.31–10.24)^a^	3.86 (1.36–10.89)^a^	0.011
≥ 30	20	602	6.09 (2.07–17.91)^a^	6.08 (2.04–18.14)^a^	0.001
HIV status					
Negative	43	3316	1.00	1.00	
Positive	23	122	14.5 (8.49–24.88)	14.6 (8.49–25.18)^a^	< 0.001
ABO blood group					
A	10	974	0.46 (0.16–1.35)		
B	16	815	0.88 (0.32–2.42)		
O	35	1426	0.83 (0.42–2.82)		
AB	5	223	1.00		
Rh blood group					
Rh Negative	4	263	1.00		
Rh Positive	62	3175	1.28 (0.46–3.66)		

*AOR* Adjusted Odds Ratio, *CI* Confidence Interval, *COR* Crude Odds Ratio

^a^Significantly Associated, HIV: Human Immunodeficiency Virus

### The trend in seroprevalence of HIV and syphilis infection

**Fig. 1:**
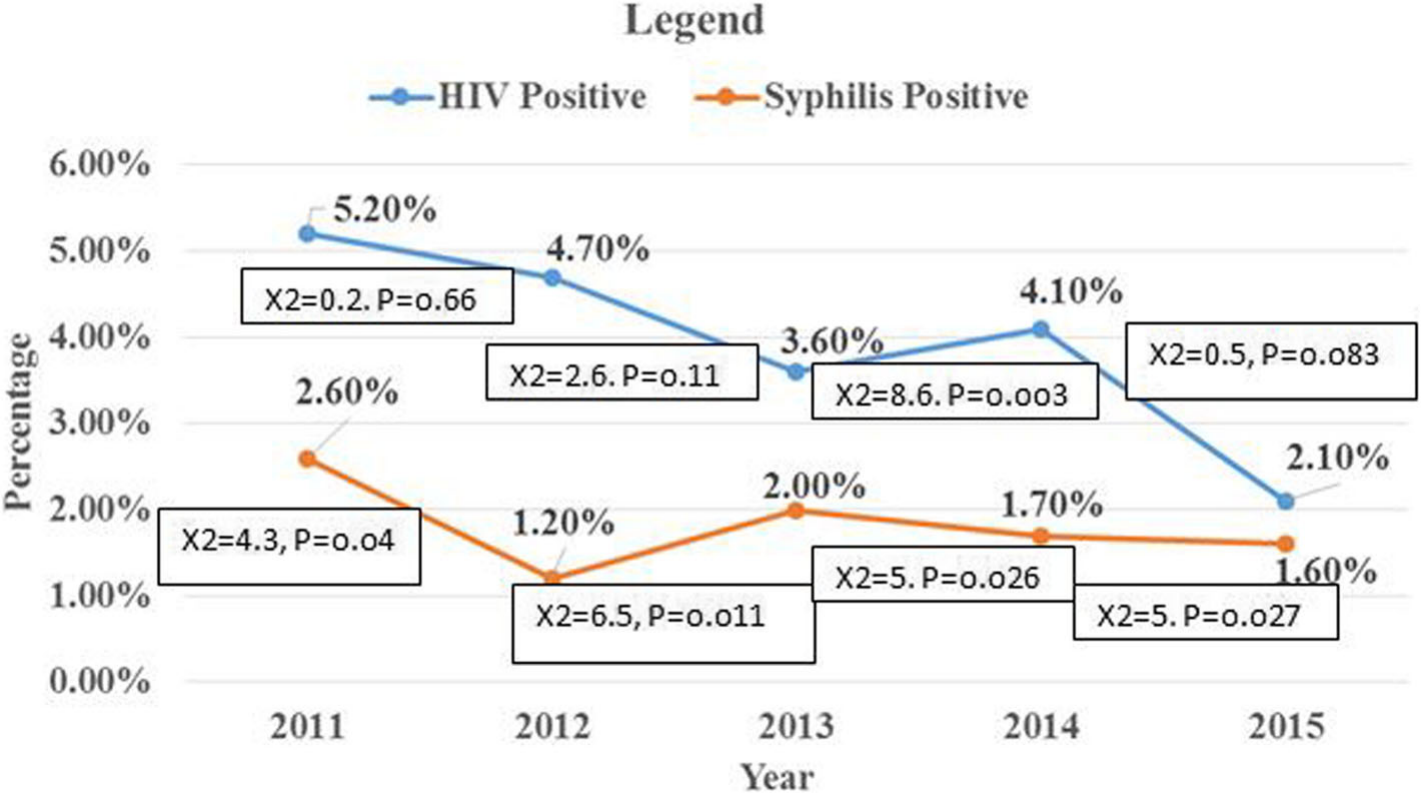
Trend prevalence of HIV and syphilis among pregnant women attending at antenatal care clinic of Gondar Family Guidance Association, northwest Ethiopia from January 2011–April 2015

The overall seroprevalence of HIV and syphilis between 2011 and 2015 among pregnant women was found to be 4.1 and 1.9%, respectively. There was a non-significant decline in trend of seroprevalence of HIV from 5.2% in 2011 to 2.1% in 2015 (χ2 = 8.03, *P*-value = 0.09), and a non significant decline in syphilis seroprevalence from 2.6% in 2011 to 1.6% in 2015 (χ2 = 4.81, P-value = 0.31). Syphilis prevalence shows a significant association in each consecutive year but in the overall trend neither was statistically significant (Fig. 1).

## Discussion

In this study, the overall prevalence of syphilis and HIV were 1.9 and 4.1% respectively and there were decline in prevalence of the HIV and syphilis from 2011 to 2015 but neither was statistically significant. The prevalence of syphilis (1.9%) is consistent with a report from Tanzania 1.6% [17], Gondar, Ethiopia 2.3% [18], Gondar, Ethiopia 1% [9]. In contrast the higher prevalence of syphilis was reported by previous studies conducted in Brazil 2.8% [19], Ethiopia, Gondar 2.9% [10], Ethiopia, Gondar 2.8% [20], Tanzania 2.5% [21], Gondar, Ethiopia 3.7% [11], and Nigeria 7.28% [22]. The variation in prevalence across studies might be attributed to the differences in method of diagnosis used, sample size, and study setting. The other factors attributed to the variation in prevalence might be due to the increased access to healthcare facilities for pregnant women for sexually transmitted disease screening at the ANC clinic [23]. Inappropriate usage of antibiotic treatment for many other infectious diseases which may lead to the advent of resistance to drug and ineffective treatment of syphilis, and re-infection from their partners, as using protective methods like condom is uncommon among married couples, they are known to have impact on the difference in seroprevalence of syphilis [24].

Significantly higher seroprevalence of syphilis was documented in the age group of 20–29 and ≥ 30 years old and HIV sero positives. This was consistent with the study conducted in Republic of Congo, that showed a high syphilis infection in the age of 35–39 years old (6%) [25] and other previous studies [9–11, 20, 26]. This higher prevalence among this age group may be attributed to the increased risk of exposure to STIs with time. In older age group, they are more likely to engage in unsafe sexual activity [27]. As a result, they may have higher rate of syphilis infection than younger aged women. The variation in prevalence across studies might be due to differences in socio-cultural diversity, socioeconomic status, prevention and control methods, societal risk factors, level of awareness about the prevention methods, and difference in laboratory methods used for diagnosis.

In the current study, the overall seroprevalence of HIV was 4.1%. This study was higher than the Ethiopian national sentinel surveillance of 2014 (2.2%) [28], and studies done in Tanzania (2%) [17], Brazil (0.3%) [19] and Nigeria (3.2%) [26]. In contrast, our study reported lower seroprevalence of HIV compared to previous studies in Nigeria (5.9%) [22], Ethiopia (6.6%) [29], Cameron (6%) [30], Ethiopia (5.4%) [31] and Ethiopia, Gondar (9.6–11.2%) [9–11]. The variation in seroprevalence across studies might be attributed to the differences in method of diagnosis used, sample size, and study setting. The other factors might be attributed to the variation in sexual behavior risk and other contributing risk factors for HIV transmission and also it may be because of the variation in socioeconomic status of the study population [32]. Evidence supported that sexually transmitted infections risk to be associated with income, since lower income is associated with less access to preventative information and healthcare, and increased used of sex for economic purpose and as a psychosocial coping mechanism [33]. Higher prevalence of HIV was seen in age group of 20–29 and ≥ 30 years old. This is similar with the 2014 Ethiopian national surveillance, where women with age group of 25–34 and 35–49 had higher HIV prevalence and the peak for HIV prevalence toward old age group [28]. This indicates that HIV seroprevalence among younger age is lower than that of the older age group. This higher prevalence among this age group might be attributed to greater knowledge about HIV prevention method is higher in younger age group than older age group, and older age group has increased risk of exposure to STD with time and in older age group they are more likely to engage in unsafe sexual activity, may not consider themselves at risk of HIV infection, as a result they may have higher prevalence of HIV than younger age women [27].

In this study the rate of co-infection was found to be 23 (0.66%). Syphilis was significantly associated with HIV infection, where HIV-infected women had higher risk of syphilis compared with HIV-non-infected women. Other similar studies reported the existence of association between HIV and syphilis in different areas and sub-populations. This co-infection rate is higher than a study in northeastern Brazil 0.05% [19] and consistent with previous studies in Gondar, Ethiopia 1% [11], 0.5% [10] but the rate of co-infection in this study is lower compared to previous study in Gondar, Ethiopia 2.2% [9]. The overall national proportion of HIV among syphilis seropositive women (4.3%) is twice that of syphilis seronegative women 2.2% [28]. This result is similar with general knowledge, for HIV and syphilis infection that share similar risk factors and mode of transmission. Syphilis infection increases the risk of transmission of HIV [4]. Hence, every pregnant woman with HIV infection should be screened for syphilis because of placental inflammation from congenital infection due to syphilis might increase the risk for perinatal transmission of HIV [34]. Reports also revealed that, there is varying strength of connotation between HIV and syphilis in various risk groups. However, none of these evidences reported whether syphilis and HIV were contracted concurrently or one infection preceded another to elucidate the causal nature of such epidemiologic interaction between HIV and syphilis.

The overall blood group distribution in this study showed that majority of the mother were blood group O followed by A with higher Rh positive rate. The prevalence of syphilis and HIV were higher in blood group O followed by B in syphilis infection and blood group A for HIV infection but not significantly associated in both infections. A study in Pakistan reported, the substantial connotation between A blood group and HIV infection whereas O blood group had no significant association with any blood transmissible infection and the study indicated that people having blood group A are more susceptible to get HIV infection [35]. Another study in India noted that blood group A negative was more susceptible to syphilis and HIV infection [36]. A similar study in Iran reported that, HIV infection was higher in blood group A but no significant association between syphilis infections with ABO and Rh blood groups [37]. Consistent with this study, the Indian study showed that there was no significant association of HIV and syphilis infection with blood group [38].

The study showed a declined prevalence of HIV infection from 45 (5.2%) in 2011 to 9 (2.1%) in 2015. This result is similar with the national HIV surveillance report [28]. It is congruent with a study in Ethiopia showing a declining trend from 8.3% in 2006 to 4.3% in 2010 [31], and a study done in Nigeria revealing a declining trend from 10.7% in 2010 to 6.8% in 2013 and 5.8% in 2014 [39]. It is also similar with the study in Zambia from 24.5% in 2002 to 21.4% in 2006 was found among young pregnant women [40]. The observed decline in HIV seroprevalence may have resulted from multiple factors including HIV control and mitigation efforts such as behavioral change communication, health education, community sensitization, widespread implementation of prevention methods, increased uptake of antiretroviral therapy, improved access to voluntary HIV counseling and testing and other interventions, over time the study participants were aware of the mode of transmission as well as the prevention method [28].

The trend in seroprevalence of syphilis infection among pregnant women showed a declining magnitude: from 23 (2.6%) in 2011 to 7 (1.6%) in 2015. This is in line with previous study in Botswana, the decreasing trend reported, from 12.4% in 1992 to 4.3% in 2003 [41], and study in Botswana demonstrated a steady decline in syphilis sero-prevalence from 2.96 to 1.15% [42] and other study in Rwanda reported, the decrease in prevalence of syphilis from 3.8% in 2002 to 2.0% in 2011 [43]. This may be an indicator for declining syphilis and HIV prevalence. It can also be evidence for safer sexual behavior as well as effectiveness of the intervention strategies implemented to reduce STDs. The limitation of this study is that the retrospective nature of the study design and the data did not include all modifiable risk factors associated with the HIV and syphilis infections.

## Conclusion and recommendation

This study showed lower HIV and syphilis prevalence in the study area compared to previous studies, but still a major public health problem. The age group 20–29, age group ≥30 years old and HIV-infection were significantly associated with syphilis infection among pregnant women. An integrated prenatal HIV and syphilis screening, and treatment is important in the study setting. Moreover, implementing focused and targeted STDs prevention strategies are advisable to decrease the magnitude of HIV and syphilis in the community. Further community-based prospective studies with larger sample size are needed to investigate the possible risk factors for HIV and syphilis infections.

## Abbreviations

ANC: Antenatal Care
AOR: Adjusted Odds Ratio
CI: Confidence Interval
COR: Crude Odds Ratio
HIV: Human Immunodeficiency Virus
MTCT: Mother to Child Transmission
PMTCT: Prevention of Mother to Child Transmission
STDs: Sexual Transmitted Diseases
STIs: Sexual Transmitted Infections
